# A novel method for high accuracy sumoylation site prediction from protein sequences

**DOI:** 10.1186/1471-2105-9-8

**Published:** 2008-01-08

**Authors:** Jialin Xu, Yun He, Boqin Qiang, Jiangang Yuan, Xiaozhong Peng, Xian-Ming Pan

**Affiliations:** 1 The Key Laboratory of Bioinformatics, Ministry of Education, China, Department of Biological Sciences and Biotechnology, Tsinghua University, Beijing, 100084, China; 2 The National Laboratory of Medical Molecular Biology, Institute of Basic Medical Sciences, Chinese Academy of Medical Sciences, and Peking Union Medical College, Chinese National Human Genome Center, Beijing 100005, China; 3 National Laboratory of Biomacromolecules, Institute of Biophysics, Academia Sinica, Beijing, China

## Abstract

**Background:**

Protein sumoylation is an essential dynamic, reversible post translational modification that plays a role in dozens of cellular activities, especially the regulation of gene expression and the maintenance of genomic stability. Currently, the complexities of sumoylation mechanism can not be perfectly solved by experimental approaches. In this regard, computational approaches might represent a promising method to direct experimental identification of sumoylation sites and shed light on the understanding of the reaction mechanism.

**Results:**

Here we presented a statistical method for sumoylation site prediction. A 5-fold cross validation test over the experimentally identified sumoylation sites yielded excellent prediction performance with correlation coefficient, specificity, sensitivity and accuracy equal to 0.6364, 97.67%, 73.96% and 96.71% respectively. Additionally, the predictor performance is maintained when high level homologs are removed.

**Conclusion:**

By using a statistical method, we have developed a new SUMO site prediction method – SUMOpre, which has shown its great accuracy with correlation coefficient, specificity, sensitivity and accuracy.

## Background

Sumoylation, a reversible post-translational modification (PTM) by the small ubiquitin-related modifier (SUMO) is essential to dozens of cellular activities, including subcellular transport, control of gross subnuclear architecture, direct and indirect effects on transcription, regulation of DNA damage recovery and replication, chromosome segregation, cell cycle progression, and competition with other ubiquitin-like modifiers (Ubls) [1-3]. Sumoylation is reportedly also a factor in various diseases and disorders, especially neural diseases, such as neuronal intranuclear inclusion disease (NIID), Alzheimer's disease (AD), and Parkinson's disease (PD) [4,5]. SUMO proteins are highly conserved across eukaryotes, and mammals express four highly conserved SUMO genes – SUMO-1, SUMO-2, SUMO-3, and SUMO-4-among which SUMO-1 has received the most attention. Yeasts express only a single SUMO gene, while plants express at least eight. However, the exact role played by such a modification – for example, positive or negative transcriptional regulation – is still unknown. Thus, more detailed information is needed on sumoylation substrates and sites.

It was still widely accepted that *ψ*KxE/D [6,7] (*ψ *represents a large hydrophobic amino acid and x represents any amino acid) is the consensus motif for SUMO-1 conjugation. However, there were many cases of sumoylation which did not occur at sites with this consensus motif. In fact, approximately 26% (69/268) of confirmed sumoylation sites contain a non-consensus motif. Although it has been reported that in some cases a short peptide containing the *ψ*KxE/D motif and a nuclear localization signal(NLS) is sufficient for SUMO-1 recognition *in vivo *[3], SUMO E3 ligases that increase the efficiency of SUMO conjugation may require more sequence information [1]. Therefore, it is necessary to focus on the exact sumoylated site and the related sequence information that may be required.

Currently, the complexities of sumoylation mechanism can not be perfectly solved by experimental approaches. Mutational analysis has been widely used in the identification of the majority of known sumoylated sites. However, while facing larger and more complex proteins, especially those with dozens of potential consensus and non-consensus sumoylation sites, mutational analysis would be labor-intensive and time-consuming. Another approach – large-scale proteomic approach is more suitable for high-throughput identification. But limited by reagent availability and the efficiency of computational peptide identification, its accuracy and stability are not so perfect. Furthermore, their current results mainly concentrate on the identification of sumoylation substrates rather than the sites [8-11]. Although Pedrioli *et al.*[12] have introduced SUMmOn, an automated theoretical pattern recognition tool that identifies sumoylated sites by detecting diagnostic PTM fragment ion series within complex MS/MS spectra, its practical sensitivity and accuracy require further validation. In this regard, computational approaches might represent a promising method to direct experimental identification of sumoylated sites. SUMOplot, for instance, is the first sumoylation site prediction tool and made a great progress. But limited by its over-concentration on data with *ψ*KxE or *ψ*KxE/D consensus motif, the prediction results may miss many non-consensus true positives. Another recent bioinformatical tool SUMOsp which applies GPS and MotifX on sumoylated site prediction, has achieved its prediction sensitivity as high as 89.12% [13]. Nevertheless, the large number of free parameters and small size of true-positive dataset may cause over-prediction. And the most appropriate performance measurement, Matthews' correlation coefficient (CC), is not so great in either prediction tool.

In the current work, SUMOpre employed a new statistical method, to predict sumoylated sites based on its adjacent amino acid subsequence. Correlation coefficient of 0.6401 was significantly higher than those in SUMOplot (0.4785) and SUMOsp (0.4873). The 5-fold cross validation/self-consistency also showed higher specificity (97.67%/97.74%) and accuracy (96.71%/96.79%), while keeping sensitivity (73.96%/74.25%) at equivalent levels to those in the two published predictors. In addition, the predictor performance was maintained when high level homologs were removed. All these results revealed that SUMOpre has a greater robustness and prediction accuracy for sumoylation site prediction. The SUMOpre web server is available on line [14].

## Results

### Effect of window length and threshold value on prediction performance

In order to derive good prediction parameters from limited experimental data, especially those with such a significantly unbalanced number of positives to negatives (approximately 1:25), it is crucial to confirm the appropriate one-side window length (*n*) and threshold value (Thd) and to realize their effects on prediction performance.

**Figure 1:**
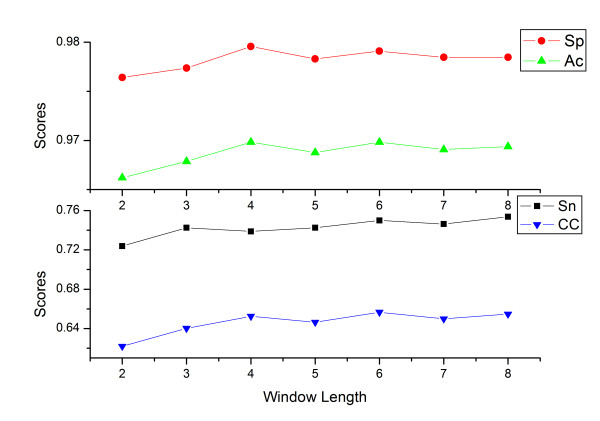
Dependence of specificity (Sp, red circle), accuracy (Ac, green triangle), sensitivity (Sn, black rectangle) and Matthews' correlation coefficient (CC, blue reversing triangle) on one-side window length (*n*).

As shown in Fig 1, assuming Thd = 0.30, the size of *n *adjacent to the Lys site had much more impact on Sn and CC than on Sp and Ac, which kept their level around 98.0% and 97.0%, respectively. As *n *increased, CC raised smoothly from 0.6218 (*n *= 2) to 0.6498 (*n *= 6) and then remained around 0.64 (*n *= 7, 8). Sn showed a similar trend, reaching its peak of 0.75 at *n *= 6. It seems reasonable to choose *n *= 6 as the default one-side window length. However, when setting *n *= 6, the number of free coefficients would increase to 228, the approximate size of the positive control dataset. Consequently, the free coefficients would directly "remember" almost all of the information without any optimization, and result in unrealistically high accuracies on current data and low accuracies on unknown protein sequences. Therefore, in order to reduce the size of free coefficients and maintain stability and applicability for both the current dataset and further predictions, we chose *n *= 3 as the default one-side window length. Based on the increasing tendency of Sn, and the smooth trends of CC, Sp and Ac, such a setting would seldom lose prediction accuracy.

**Figure 2:**
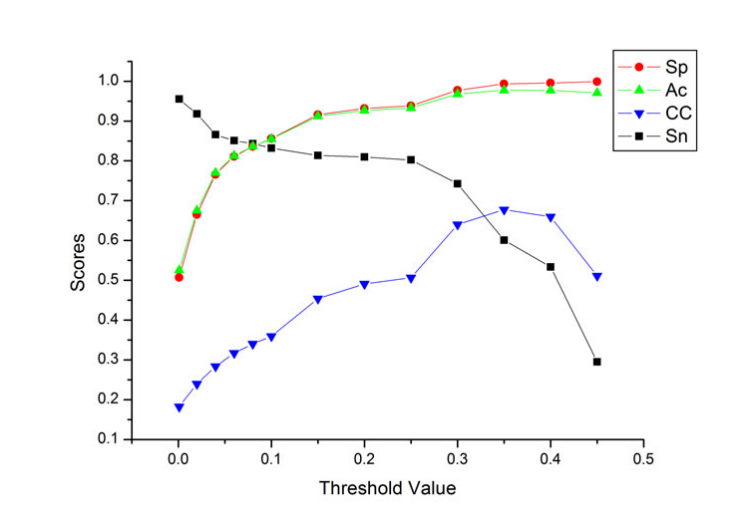
Dependence of specificity (Sp, red circle), accuracy (Ac, green triangle), sensitivity (Sn, black rectangle) and Matthews' correlation coefficient (CC, blue reversing triangle) on the threshold value (Thd).

The dependences of the four measuring parameters corresponding to Thd were shown in Fig. 2. As Thd increased, CC smoothly ascended and reached its peak at 0.6774 (Thd = 0.35), while Sn initially showed a smooth descent before declining dramatically when Thd≥0.25. Additionally, Sp and Ac increased sharply at a similar rate, and then remained above 97% and 99%, respectively, at Thd≥0.3. In this paper, 0.3 was set as the default cut-off Thd, with user-defined values (0.2–0.4) in the web server. Choosing lower cut-off Thd, such as Thd = 0.2, would facilitate a prediction with much higher sensitivity (80.97%), but lower CC, Sp and Ac. On the other hand, setting higher Thd, such as 0.35, would maintain much higher robustness with CC = 0.6774, but relatively low sensitivity. Users can choose different cut-off Thd according to their experimental designs and prediction expectations.

### Stability of SUMOpre

**Figure 3:**
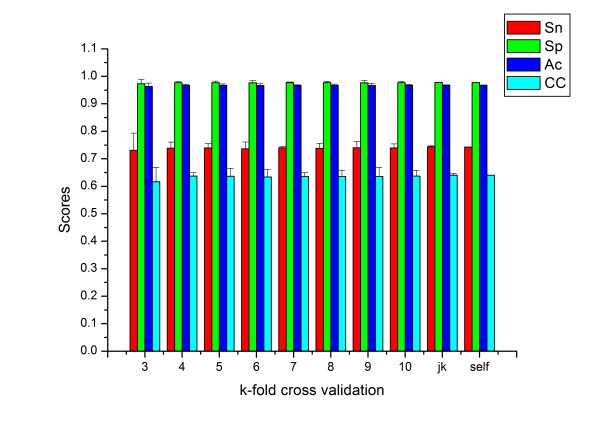
Performance of self-consistency, K-fold (3-, 4- ... 10-fold) cross-validation and jack-knife validation. Sn, Sp, Ac, CC in each validation are represented with red, green, blue and cyan bars, respectively.

In order to test the stability of the SUMOpre method, we employed three strategies on the same dataset: k-fold cross-validation, jack-knife validation and self-consistency tests. The test performances were shown in Fig. 3. Sn, Sp, Ac and CC (represented by red, green, blue and cyan bars, respectively) corresponding to the k-fold (3≤k≤10) cross-validation were found in the first eight columns, while both jack-knife and self-consistency test results were in the last two columns. Interestingly, these prediction performances were all robust with relatively small standard error among these tests. CC, for instance, maintained a stable level around 0.6401 in all three tests with only a small standard error.

One vital factor that could result in misleadingly high prediction performance and possibly influence prediction stability is sequence homology in training dataset. In fact, if the training data and test data are identical or highly homologous, for example, at a sequence identify level higher than 30%, the memorization effects in the self-consistency tests cannot be completely removed. Consequently, the prediction accuracy could be misleadingly high [15]. Here, NCBI BLASTCLUST software was used to filter out highly homologous sequences, after which SUMOpre was rerun to evaluate differences between the original dataset and the low-homology dataset. Table 1 lists the prediction performances of the low homology datasets. CC persisted at a level of 0.65 after the shift to a lower homology level. Simultaneously, Sp and Ac also kept their traces in both jack-knife and self-consistency validation, regardless of the similarity and minimum length coverage cut-off. With decreases in minimum length coverage cut-off value (from 0.8 to 0.6 and 0.4), 19, 40 and 51 sequences were filtered out, and Sn in jack-knife validation decreased smoothly from 70.34% to 66.03% and 64.02%, respectively. Nevertheless, the fact that even with the filtering of approximately 1/3 (51/159) of homologous sequences, the tiny variety on performances is shown by Sn with a 10% decrease, sufficiently suggested the robustness of SUMOpre and its database.

**Table 1:** Performance of SUMOpre using datasets filtering out highly homologous sequences.

Dataset	Similarity^a^	Coverage^b^	size	Method^c^	CC	Sn (%)	Sp (%)	Ac (%)
Dataset 1	0.3	0.4	108	self	0.6782	64.02	99.13	97.80
				jk	0.6364	60.85	98.94	97.50
Dataset 2	0.3	0.6	119	self	0.6500	66.03	98.67	97.41
				jk	0.6061	63.64	98.35	97.01
Dataset 3	0.3	0.8	140	self	0.6520	70.34	98.33	97.24
				jk	0.5983	66.53	97.95	96.72
All data	-	-	159	self	0.6401	74.25	97.74	96.79
			(268 sites)	jk	0.5911	70.90	97.30	96.23

^a ^Similarity threshold used in NCBI BLASTCLUST was set as 0.3;

^b ^Minimum length coverage threshold used in NCBI BLASTCLUST;

^c ^Validation strategies: self, self-consistency test; jk, jack-knife validation

To further illustrate the robustness of SUMOpre in regard to threshold-independent performance, receiver operating characteristic (ROC) curves of self-consistency, jack-knife validation and 5-fold cross validation were provided (see Additional file 1). After comparisons with SUMOsp [13], both the ROC curves and the areas under the ROC curves (AUC) again obviously imply the robustness of SUMOpre.

### Comparison of SUMOpre with SUMOplot and SUMOsp

In order to comprehensively compare the prediction performance of SUMOpre with SUMOsp and SUMOplot, two separate datasets were utilized. The first dataset contained all 159 training sequences for both training and testing, The other dataset used 144 substrate sequences obtained before December 10, 2005 (employed by SUMOsp [13] as training and testing data) for training plus 15 new sequences reported later for testing (not included in SUMOplot or SUMOsp). Since the Jack-knife validation could not be performed again for the other two predictors, we submitted the substrate sequence into these tools and adopted the self-consistency performance of all three tools for comparison. The two levels of stringency in SUMOplot were denoted as high (motifs with high probability) and all (all predictions) just as defined on the web site [16]. The cut-off of SUMOsp was defined as 4 and 18 [13,17]. In Table 2, using all 159 proteins for training and testing, the CC, Sn, Sp and Ac of SUMOpre with a Thd of 0.3 are 0.6401, 74.25%, 97.74% and 96.79%, while the CC, Sn, Sp and Ac with a Thd 0.2 are 0.4908, 80.97%, 93.18% and 92.68%, respectively. In contrast to SUMOplot and SUMOsp, CC, the most important measuring parameter for the biased dataset, is higher or at least equal to any level of the other two predictors when SUMOpre is at a level of 0.2. Additionally, CC at the 0.3 level of SUMOpre is significantly higher (0.6401) than the top level of SUMOsp (0.4873) and SUMOplot (0.4785). More importantly, Sp and Ac at either level of SUMOpre (97.74% & 96.79%) are also much higher than those of SUMOsp (93.05% & 92.56%) and SUMOplot (93.43%&92.79%), with equivalent sensitivity at 74.25%.

**Table 2:** Performance comparisons using the whole dataset for training and testing.

Predictor	Threshold	CC	Sn (%)	Sp (%)	Ac (%)
SUMOpre*	0.30	0.6401	74.25	97.74	96.79
	0.20	0.4908	80.97	93.18	92.68
SUMOsp	18	0.4873	80.97	93.05	92.56
	4	0.3134	86.57	80.03	80.30
SUMOplot	High	0.4785	77.61	93.43	92.79
	All	0.3123	85.45	80.46	80.66

*The self-consistency test was used as the testing strategy.

After shifting the training dataset to the 144-protein dataset used by SUMOplot, and the testing dataset to the 15-protein dataset with sites newly identified, the performance of SUMOpre was much more robust and accurate than that of other predictors. Because the testing dataset completely differs from the training group, prediction performance comprehensively represented their actual robustness. For instance, as shown in Table 3, CC, Sp and Ac at the 0.35 level of SUMOpre are also considerably higher (0.6566, 99.51% and 97.49%, respectively) than those in the upper levels of SUMOsp and SUMOplot, while Sn of SUMOpre is definitely equal to that of SUMOsp and SUMOplot. Thus, SUMOpre provides better robustness, specificity and accuracy while retaining a similar level of sensitivity.

**Table 3:** Performance comparisons using the 144-protein dataset for training and 15-protein dataset for testing.

Predictor	Threshold	CC	Sn (%)	Sp (%)	Ac (%)
SUMOpre*	0.35	0.6566	53.57	99.51	97.49
	0.20	0.3651	60.71	92.45	91.05
SUMOsp	18	0.3726	60.71	92.78	91.37
	4	0.2361	67.86	79.97	79.43
SUMOplot	High	0.3503	57.14	92.78	91.21
	All	0.2097	64.29	78.82	78.18

*The self-consistency test was used as the testing strategy.

### Use of Web service

**Figure 4:**
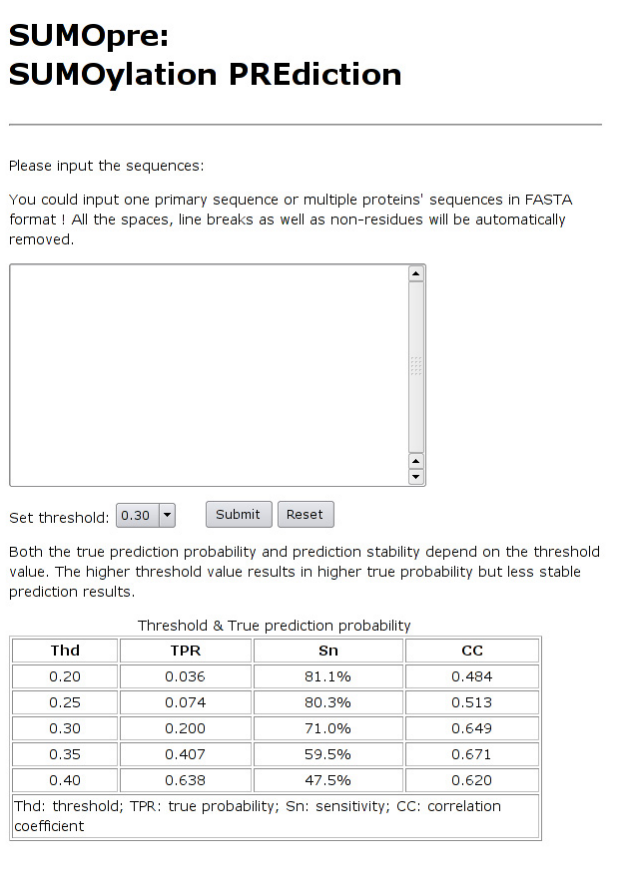
The prediction page from the SUMOpre web server.

The program SUMOpre was implemented in C++, and its Web server [14] has been developed in an easy-to-use manner. Users can visit SUMOpre web server (Fig. 4), submit the protein sequences in FASTA format into the text box, choose the proper Thd and run the program. Probability for true positive prediction (TPR) corresponding to different threshold choices are provided in the table below as a reference for various prediction expectations. According to the threshold value, the prediction result includes the potential sumoylated sites, scores and probability of true positive prediction.

## Discussion

We have successfully developed a highly robust sumoylated site prediction tool with the use of statistical methods. In order to avoid overtraining due to the limited experimental data, the predictor performance is maintained when high level homologs are removed and using as few as possible fitting parameters. A predictor with too many free coefficients would directly "remember" almost all of the information without any optimization, and result in unrealistically high accuracies on current data and low accuracies on unknown protein sequences. Thus, SUMOpre provides better robustness, specificity and accuracy while retaining a similar level of sensitivity as other prediction methods. Furthermore, considering the highly unbalanced training data (the negative training dataset is approximately 25 times large of the positive dataset), the main parameter for assessment of predictive performance should be the Matthews' correlation coefficient, *CC*, that of SUMOpre is significantly higher (0.6401) than the top level of SUMOsp (0.4873) and SUMOplot (0.4785).

Since about 74% of confirmed sumoylation sites in the training data contain a consensus motif, the free coefficients obtained by training would be optimized to "remember" more information for the consensus motif. The prediction specificity of SUMOpre, Sp, is 0.89 for consensus sites and 0.25 for non-consensus sites, implying that the prediction of non- consensus sites is fairly hard.

### Sumoylation mechanism significantly depends on sequence information

Why was SUMOpre able to perform so well by simply utilizing sequence information? It is mainly due to the corresponding sumoylation mechanism that is heavily dependent on sequence information. SUMO is conjugated to target proteins by an enzymatic cascade involving a SUMO activating enzyme (E1), a SUMO conjugating enzyme (E2), and typically a SUMO ligase (E3). SUMO proteins are activated by the heterodimeric E1 AOS1-UBA2 that use the E2 UBC9 for conjugation. There are currently three types of known E3s for the SUMO proteins – RanBP2 (Ran-binding protein-2), PIASs family and Pc2 (Polycomb 2 homolog). These three types of enzymes have distinct subcellular localizations and mediate the modification of specific substrates [1,18]. Furthermore statistic over the 57 sites with identified PDB structures, there are only 3 sites buried in protein interior (K99 in 1JI7 with 17% exposed, K347 in 1AM9 with 12% exposed and K447 in 1U0J with 7% exposed) and all other 54 sites exposed on the protein surface. This may reflect the fact that UBC9 makes direct contact with substrates and has sequence preference. In contrast to more than one thousand protein kinases and their complicated phosphorylation recognition and modification systems with dissimilar site preferences [19], direct SUMO recognition on the single Lys site merely relies on limited factors: three enzymes and other elements such as subcellular localization or appropriate presentation of the sequence on the substrates [1,3]. Without various enzymes and their complex recognition mechanisms and other factors, motif recognition based solely on sequence information could be sufficient for sumoylation prediction.

As discussed by Matunis & Pickart [20], sumoylation is frequently site specific, which may refer to the maximum benefit from reduced entropy if the reacting lysine residue is forced into a catalytically favorable orientation. Furthermore, the performance of SUMOpre, based merely on sequence information from known sumoylated sites, supports the suggestion of a sequence-dependent recognition and modification mechanism. In fact, using a dataset with 268 sumoylation sites (that includes 69 with non-consensus motifs) and 6,361 non-reported sumoylation Lys sites (including 210 sites with consensus motifs), we have achieved a powerful predictor with CC = 0.6401 and sensitivity = 74.25%. All findings indicate that sequence information, especially the close proximity of a Lys to sequence information, is an essential factor impacting the specificity of SUMO recognition and modification.

## Conclusion

By using a statistical method, we have developed a new SUMO site prediction method – SUMOpre, which has shown its great accuracy with correlation coefficient, specificity, sensitivity and accuracy equal to 0.6364, 97.67%, 73.96% and 96.71% in 5-fold cross validation, respectively. Due to the full consideration on both consensus *ψ*KxE/D and non-consensus motif, our method achieved greater robustness (0.15 higher correlation coefficients) than other published predictors. Furthermore, our prediction accomplishment based on protein sequence supports the suggestion of a sequence-dependent recognition and modification mechanism.

## Methods

### Dataset

PubMed was searched with keywords 'SUMO' and 'sumoylation' and obtained 268 unambiguously experimentally defined sumoylation sites in 159 proteins from 710 research articles published online before Aug. 10, 2006. Their primary sequences have also been extracted from Swiss-Prot/TrEMBL database [21]. In those 159 protein sequences, there are a total of 6,629 lysine (Lys) sites, including 268 experimentally identified sumoylated sites used as positive training data, and 6,361 non-reported sumoylation Lys sites.

In order to compare prediction performance with other two published predictors, the dataset was divided into two subsets for performance comparison among the three predictors. One 144-protein set included proteins within 240 experimentally identified sumoylated sites reported before December 10, 2005, and utilized in SUMOsp. The other 15-protein one contained proteins within 28 experimentally identified sumoylated sites that were reported after December 10, 2005. The other K sites not reportedly sumoylated were collected as negative training datasets for both subsets (see Additional file 2 and 3).

### Algorithms

We are interested in predicting the sumoylation state of residue Lys on position i, *ω*_*i *_(sumoylated or non-sumoylated), based on knowledge of the amino acid subsequence window, {A_*i*_}, of a restricted size (2*n*+1) (*n*: one-side window length) symmetric about position i. Positions within the window are indexed by j (j = 1, 2,..., 2n+1), with j = *n*+1 equaling to position i. Thus, given a subsequence, the conditional probability of the sumoylation states (*ω*_*i*_):

(1) $$\mathrm{Pr}(\omega_{i}|{\text{A}}_{i})=\frac{\mathrm{Pr}(A_{i},\omega_{i})}{\mathrm{Pr}(A_{i})}=\mathrm{Pr}(\omega_{i})=\frac{\mathrm{Pr}(A_{i}|\omega_{i})}{\mathrm{Pr}(A_{i})}$$

Here Pr(*ω*_*i*_) denotes the probability of sumoylation state *ω*_*i *_of the central residue Lys; Pr(A_i_) represents the probability of subsequence A_i_; Pr(A_i_, *ω*_*i*_) means the probability of both subsequence A_i _and sumoylation state *ω*_*i *_of central residue Lys occur; and Pr(A_i_|*ω*_*i*_) is the conditional probability of the subsequence Ai given the sumoylation state. If assuming that the 2n positions in the window independently, then the subsequence probability could be obtained by multiplying the single-position probability over the window [22].

$$\begin{array}{c} \mathrm{Pr}(A_{i})=\prod_{\begin{array}{c} j=1\\ j\neq n+1 \end{array}}^{2n+1}\mathrm{Pr}(R_{j})\\ \mathrm{Pr}(A_{i}|\omega_{i})=\prod_{\begin{array}{c} j=1\\ j\neq n+1 \end{array}}^{2n+1}\mathrm{Pr}(R_{j}|\omega_{i})=\prod_{\begin{array}{c} j=1\\ j\neq n+1 \end{array}}^{2n+1}\mathrm{Pr}(R_{j})\frac{\mathrm{Pr}(\omega_{i}|R_{j})}{\mathrm{Pr}(\omega_{i})} \end{array}$$

Here R_j _represents residue type R on position j. Pr(*ω*_*i*_|R_j_) is the conditional probability of the sumoylation state *ω*_*i *_for position j given that it is occupied by residue type R. So we can simplify Eq. (1) as following:

(2) $$\mathrm{Pr}(\omega_{i}|A_{i})=\mathrm{Pr}(\omega_{i})\prod_{\begin{array}{c} j=1\\ j\neq n+1 \end{array}}^{2n+1}\frac{\mathrm{Pr}(\omega_{i}|R_{j})}{\mathrm{Pr}(\omega_{i})}$$

Eq. (2) is equivalent to the secondary structure prediction model of GOR I [22]. Since the assumption of position independently was not so solid, GOR IV [23] uses pairwise information over all possible paired positions in the window. As the experimental sumoylation data are not large enough to provide sufficient data, we simply modified the model as following:

(3) $$\mathrm{Pr}(\omega_{i}|A_{i})=\mathrm{Pr}(\omega_{i})\prod_{\begin{array}{c} j=1\\ j\neq n+1 \end{array}}^{2n+1}{\left(\frac{\mathrm{Pr}(\omega_{i}|R_{j})}{\mathrm{Pr}(\omega_{i})}\right)}^{\alpha (R_{j})}$$

After taking the natural logarithm on both sides of Eq.(3), we obtain:

(4) $$\ln(\mathrm{Pr}(\omega_{i}|A_{i}))=\sum_{\begin{array}{c} j=1\\ j\neq n+1 \end{array}}^{2n+1}\alpha (R_{j})\ln(\frac{\mathrm{Pr}(\omega_{i}|R_{j})}{\mathrm{Pr}(\omega_{i})})+\ln(\mathrm{Pr}(\omega_{i}))$$

In Eq. (4), both *α*(R_j_) and $\ln(\frac{\mathrm{Pr}(\omega_{i}|R_{j})}{\mathrm{Pr}(\omega_{i})})$ are position- and residue type- dependent parameters. So we define:

$$\theta_{j}(\omega_{i}|R_{j})≜\alpha (R_{j})\ln(\frac{\mathrm{Pr}(\omega_{i}|R_{j})}{\mathrm{Pr}(\omega_{i})})$$

For convenience, we move a constant from the left to the right side and define the left side as a value, I(*ω*_*i*_), which represents the sumoylation states by 1 (sumoylated) or 0 (non-sumoylated). Rewriting the formula gives:

(5) $$I(\omega_{i})=\sum_{\begin{array}{c} j=1\\ j\neq n+1 \end{array}}^{2n+1}\theta_{j}(\omega_{i}|R_{j})+C$$

Here, *θ*_j_(*ω*_i_| R_j_) is a position-dependent 20-demisional vector for 20 types of amino acids. C is a constant. If the subsequences in the window are DAMKNEC, for example, the equation for the sumoylated state of Lys is:

1.0 = *θ*_1_(D) + *θ*_2_(A) + *θ*_3_(M) + *θ*_5_(N) + *θ*_6_(E) + *θ*_7_(C) + C

That for the non-sumoylated state is:

0 = *θ*_1_(D) + *θ*_2_(A) + *θ*_3_(M) + *θ*_5_(N) + *θ*_6_(E) + *θ*_7_(C) + C

Eq. (5) is a linear equation. All the coefficients could be determined by the data in the training dataset using Multiple Linear Regression method (MLR) to minimize the sum of the square of deviation between the left and right side of the equation. In the practical MLR process, one component should be left out from the 20-demisional vector *θ*_j_, and the number of free coefficients is 2 × *n *× 19. In the above example with *n *= 3, there are 114 (2 × 3 × 19) free coefficients to be determined.

### NCBI BLASTCLUST filter for highly homologous sequences

NCBI BLASTCLUST was employed to filter out highly homologous protein sequences from the original dataset [15]. BLASTCLUST automatically and systematically clusters protein sequences based on pairwise matches found using the BLAST algorithm. Similarity threshold and minimum length coverage are two crucial parameters for filtering out highly homologous sequences: the former was set as a BLAST score density, while the later restricted the minimum percent for pairwise coverage. The similarity threshold was set at 0.3. Due to different values of minimum length coverage of the 159 protein sequences, proteins were grouped in one cluster if they shared greater similarity and larger minimum length coverage than the corresponding thresholds. Only one protein in each cluster was chosen to establish new low-homology training and test datasets, while the remaining protein sequences were filtered out.

### Accuracy measures

All reported results were based on a window of length *n *residues, symmetrically located about the residue under consideration. Considering the highly unbalanced training data (the negative training dataset is approximately 25 times large of the positive dataset), the main parameter for assessment of predictive performance was the Matthews' correlation coefficient, *CC*, which can be calculated as follows:

$$CC=\frac{\left(TP\times TN)-(FN\times FP\right)}{\sqrt{\left(TP+FN)\times (TN+FP)\times (TP+FP)\times (TN+FN\right)}}$$

Where *TP *is the number of positive cases that were correctly predicted; *TN *is the number of negative cases that were correctly rejected;*FP *is the number of over-predicted cases; and *FN *is the number of under-predicted cases.

Additionally, other general parameters, used in most other studies, were applied [13]. They were sensitivity (*Sn*), specificity (*Sp*) and accuracy (*Ac*), defined as follows:

$$\begin{array}{l} \begin{array}{cc} Sn=\frac{TP}{TP+FN}, & Sp=\frac{TN}{TN+FP}, \end{array}\\ Ac=\frac{TP+TN}{TP+FP+TN+FN}, \end{array}$$

### Self-consistency, K-fold cross-validation and Jack-knife tests

Predictive quality was examined with three approaches, one based on the re-substitution test and the other two upon k-fold cross-validation.

#### Self-consistency test

The sumoylation state for each motif in the entire dataset is predicted using the rules derived from the same dataset.

#### K-fold cross-validation

The dataset was randomly divided into k subsets. Each time, one of the *k *subsets was used as the test set and the other *k*-1 subsets were assembled to form a training set.

#### Jack-knife (Leave-one-out cross validation)

An extreme validation deduced from k-fold cross validation with k equal to N, the number of data points in the set. It means that N separate times, the function approximator was trained on all the data except for the point being predicted.

## List of abbreviations

PTM: post-translational modification; SUMO: small ubiquitin-related modifier; *n*: one-side window length; Thd: threshold value; CC: Matthews' correlation coefficient; Sn: sensitivity; Sp: specificity; Ac: accuracy; ROC, receiver operating characteristic curve; MLR: Multiple Linear Regression method.

## Authors' contributions

JX carried out the data analysis and interpretation, developed computer programs, and wrote the manuscript. YH conceived the data analysis and developed the web server. BQ and JY conceived the idea and revised the manuscript critically for important intellectual as well as professional content. XP and XMP conceived and coordinated the project, guided its conception and design, helped in interpretation of data, refined the drafted manuscript and gave overall supervision to the project. Especially, XMP also made a critical contribution on the computer programs. All authors read and approved the final manuscript.

## Supplementary Material

Additional file 1 The Receiver Operating Characteristic (ROC) curves of 5-fold cross validation (blue diamond), self-consistency (pink rectangle), and jack-knife validation (yellow triangle) tests. Click here for file

Additional file 2 The reported sumoylated protein dataset List. The reported sumoylated protein list, including protein access number in UniprotKB, sumoylated sites, reference Pubmed ID and PDB information if available. Click here for file

Additional file 3 The reported sumoylated protein sequences. The full-length protein sequences mentioned in Dataset List. Click here for file

## References

[B1] Hay RT (2005). SUMO: a history of modification. Mol Cell.

[B2] Kroetz MB (2005). SUMO: a ubiquitin-like protein modifier. Yale J Biol Med.

[B3] Seeler JS, Dejean A (2003). Nuclear and unclear functions of SUMO. Nat Rev Mol Cell Biol.

[B4] Dorval V, PE F (2006). Small ubiquitin-like modifier (SUMO) modification of natively unfolded proteins tau and alpha-synuclein. J Biol Chem.

[B5] Shinbo Y, Niki T, Taira T, Ooe H, Takahashi-Niki K, Maita C, Seino C, Iguchi-Ariga SM, Ariga H (2006). Proper SUMO-1 conjugation is essential to DJ-1 to exert its full activities. Cell Death Differ.

[B6] Sampson DeborahA, Wang Min, Matunis MJ (2001). The Small Ubiquitin-like Modifier-1 (SUMO-1) Consensus Sequence Mediates Ubc9 Binding and Is Essential for SUMO-1 Modification. J Biol Chem.

[B7] Rodriguez ManuelS, Dargemont Catherine, Hay RT (2001). SUMO-1 Conjugation in Vivo Requires Both a Consensus Modification Motif and Nuclear Targeting. J Biol Chem.

[B8] Denison C, Rudner AD, Gerber SA, Bakalarski CE, Moazed D, Gygi SP (2005). A proteomic strategy for gaining insights into protein sumoylation in yeast. Mol Cell Proteomics.

[B9] Gocke CB, Yu H, Kang J (2005). Systematic identification and analysis of mammalian small ubiquitin-like modifier substrates. J Biol Chem.

[B10] Hannich JT, Lewis A, Kroetz MB, Li SJ, Heide H, Emili A, Hochstrasser M (2005). Defining the SUMO-modified proteome by multiple approaches in Saccharomyces cerevisiae. J Biol Chem.

[B11] Rosas-Acosta G, Russell WK, Deyrieux A, Russell DH, Wilson VG (2005). A universal strategy for proteomic studies of SUMO and other ubiquitin-like modifiers. Mol Cell Proteomics.

[B12] Pedrioli PG, Raught B, Zhang XD, Rogers R, Aitchison J, Matunis M, Aebersold R (2006). Automated identification of SUMOylation sites using mass spectrometry and SUMmOn pattern recognition software. Nat Methods.

[B13] Xue Y, Zhou F, Fu C, Xu Y, Yao X (2006). SUMOsp: a web server for sumoylation site prediction. Nucleic Acids Research.

[B14] SUMOpre web server. http://spg.biosci.tsinghua.edu.cn/service/sumoprd/predict.cgi.

[B15] Wang ZX, Yuan Z (2000). How good is prediction of protein structural class by the component-coupled method?. Proteins.

[B16] SUMOplot web server. http://www.abgent.com/doc/sumoplot.

[B17] SUMOsp web server. http://bioinformatics.lcd-ustc.org/sumosp.

[B18] Welchman RL, Gordon C, Mayer RJ (2005). Ubiquitin and ubiquitin-like proteins as multifunctional signals. Nat Rev Mol Cell Biol.

[B19] Manning G, Whyte DB, Martinez R, Hunter T, Sudarsanam S (2002). The protein kinase complement of the human genome. Science.

[B20] Matunis MJ, Pickart CM (2005). Beginning at the end with SUMO. Nat Struct Mol Biol.

[B21] Swiss-Prot/TrEMBL database. http://cn.expasy.org.

[B22] Garnier J, Osguthorpe DJ, Robson B (1978). Analysis of the accuracy and implications of simple methods for predicting the secondary structure of globular proteins. J Mol Biol.

[B23] Garnier J, Gibrat JF, Robson B (1996). GOR method for predicting protein secondary structure from amino acid sequence. Methods Enzymol.

