# Supplementary figures and images for: A novel method for high accuracy sumoylation site prediction from protein sequences
Source: BMC Bioinformatics. 2008 Jan 8;9:8. doi: 10.1186/1471-2105-9-8 (PMC2245905; doi:10.1186/1471-2105-9-8)

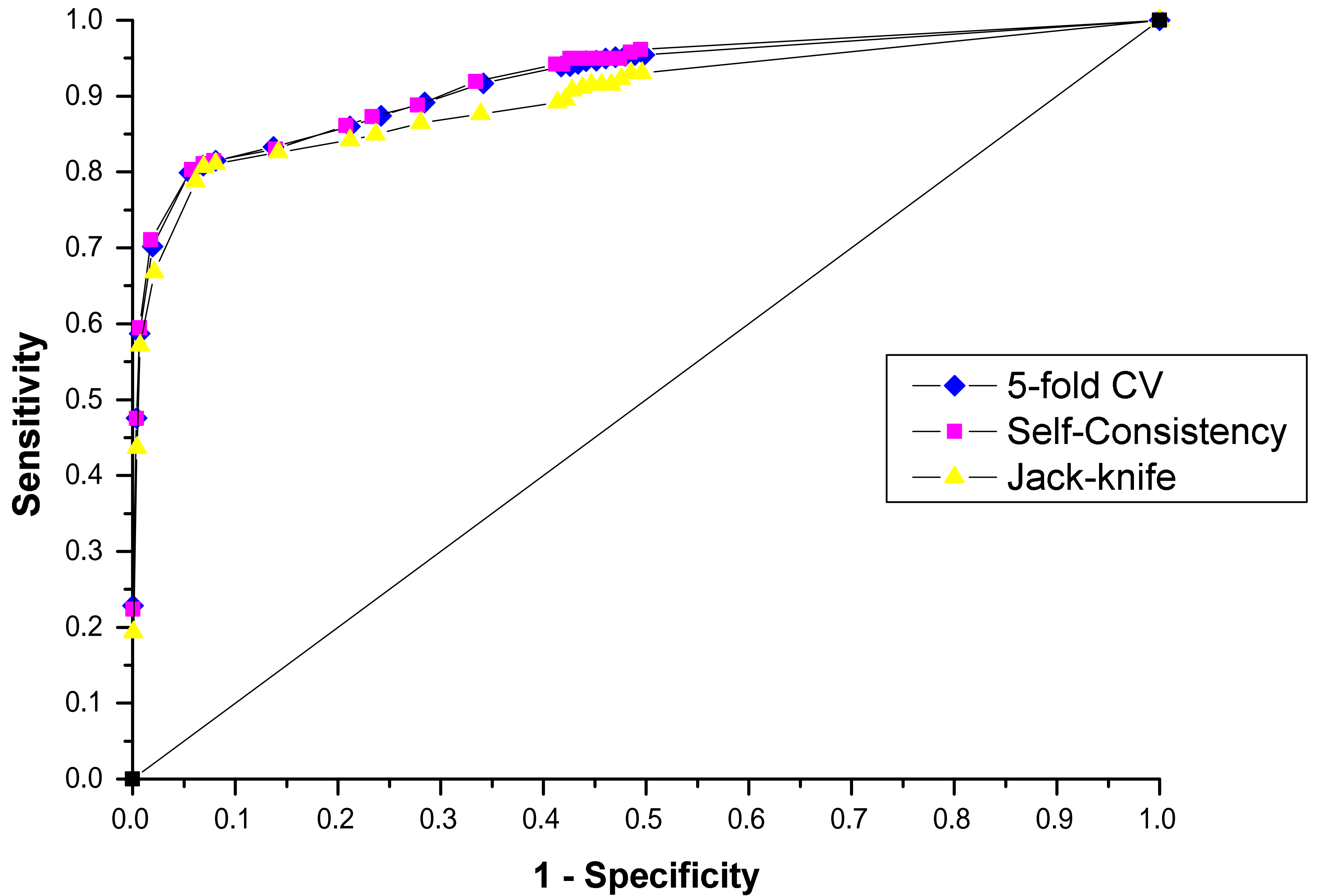

Supplement: Additional file 1 — The Receiver Operating Characteristic (ROC) curves of 5-fold cross validation (blue diamond), self-consistency (pink rectangle), and jack-knife validation (yellow triangle) tests. [file 1471-2105-9-8-S1.pdf]
