# Supplementary material for: A novel method for high accuracy sumoylation site prediction from protein sequences
Source: BMC Bioinformatics. 2008 Jan 8;9:8. doi: 10.1186/1471-2105-9-8 (PMC2245905; doi:10.1186/1471-2105-9-8)
Supplement: Additional file 2 — The reported sumoylated protein dataset List. The reported sumoylated protein list, including protein access number in UniprotKB, sumoylated sites, reference Pubmed ID and PDB information if available. [file 1471-2105-9-8-S2.pdf]

SUMOpre Training and Testing Positive Database

Protein acc. a)	SUMO Pos. b)	Ref. PMID c)	PDB acc. d)	Range e)	PDB Stru. f)
144 proteins within 240 sites g)					
Q9H3D4	588	15539951	1RG6	540-610	H
Q9H3D4	676	15539951<br>15611636			
O56136	84	15527853			
O56136	447	15527853	1U0J	225-490	C
P19544	73	15520190			
P19544	177	15520190			
O75030	289	15507434	1AN4	HSSP	
O75030	423	15507434	1AN4	HSSP	
P19532	330	15507434	1AN4	HSSP	
P19484	347	15507434	1AM9	HSSP	
P37231	107	15229330<br>15507114			
Q02447	120	12419227	1SP1	HSSP	
Q02447	551	15247228<br>15494207	1SP1	HSSP	
Q16665	391	15465032			
Q16665	477	15465032			
P46060	524	15355965	1Z5S	418-587	C
Q14191	496	15355988			
P43694	365	15337742	1GNF	HSSP	
Q08211	76	15312759	1UIL	4-89	H
Q08211	120	15312759			
P36508	411	15280358	1SP2	HSSP	
Q03188	534	15272016			
Q03188	721	15272016			
Q03188	746	15272016			
P63279	153	15272016	1A3S	1-158	O
P19419	230	14992729<br>15210726			
P19419	249	14992729<br>15210726			
P19419	254	15210726			
Q9Y4L2	244	15208321	1TF3	HSSP	
Q9Y4L2	263	15208321	1TF3	HSSP	
Q13285	119	15192080<br>15192092<br>15713642			

Q13285	194	15192080<br>15192092<br>15713642			
P15976	137	15173587	1GNF	HSSP	
P15873	127	12226657<br>15121847	1PLQ	1-258	N
P15873	164	12226657<br>15121847<br>15542864	1PLQ	1-258	C
Q05193	376	15123615			
Q60591	684	15117942	1A02	HSSP	
Q60591	897	15117942	1A02	HSSP	
P41212	99	12626745<br>15107848	1JI7	47-124	C
O13066	517	15094046	1KPS	HSSP	
O60812	237	15082759	1WF2	2-92	H
P42858	6	15064418			
P42858	9	15064418			
P42858	15	15064418			
Q13485	113	12621041<br>12740389<br>15028714	1DD1	285-552	C
Q13485	159	12621041<br>12740389<br>14514699<br>15028714<br>15637079	1DD1	285-552	C
Q03933	82	11278381<br>15023536	3HTS	HSSP	
P78347	221	15016812	1Q60	731-823	H
P78347	240	15016812	1Q60	731-823	H
P78347	456	15016812	1Q60	731-823	H
P78347	991	15016812	1Q60	731-823	H
Q9BYV9	202	15060166	1JNM	HSSP	
Q9BYV9	276	15060166	1JNM	HSSP	
Q9BYV9	421	15060166	1JNM	HSSP	
Q9BYV9	580	15060166	1JNM	HSSP	
O00429	38	14972687			
Q9Y6K9	277	14651848	1G8X	Mod	
Q9Y6K9	309	14651848	1G8X	Mod	
Q9UPW6	233	14701874			
Q9UPW6	350	14701874	1WIZ	350-437	H
P06536	297	14663148			

P06536	313	14663148			
P25963	21	14613580<br>9069263	1IKN	67-302	O
P42224	703	12855578<br>14596924<br>15761017<br>16857984	1YVL	1-683	C
P43354	91	14559918			
P43354	577	14559918	1OVL	328-598	C
Q05516	242	14527952			
Q9NS56	560	14516784	1CHC	HSSP	
P08235	89	14500761			
P08235	399	14500761			
P08235	428	14500761			
P08235	494	14500761			
P08235	953	14500761	1Y9R	731-984	C
Q05397	152	14500712			
P06401	388	12529333<br>12943706<br>12114521			
Q09472	1020	12718889<br>12887893<br>15632193			
Q09472	1024	12718889<br>12887893<br>15632193			
P17676	174	12810706			
P11831	147	12788062	1HBX	132-223	C
Q13363	428	12679040<br>12769861			
P32457	4	12149243<br>12761287			
P32457	11	12149243<br>12761287			
P32457	30	12149243<br>12761287			
P32457	63	12149243<br>12761287<br>15542864			
P32457	287	12761287			
P32457	443	12761287			
P32457	465	12761287			
Q12216	438	12761287			

Q12216	446	12761287			
Q99497	130	12761214<br>15976810	1J42	1-189	C
P23769	222	12750312			
P23769	389	12750312			
Q924A0	297	12727872	1JPW	HSSP	
Q00613	298	11514557<br>12646186<br>12665592	1HKT	HSSP	
P10242	503	12631292<br>16162816	1MBE	HSSP	
P10242	527	12631292<br>16162816	1MBE	HSSP	
P36956	123	12615929			
P36956	418	12615929			
Q12772	464	12615929			
P16220	285	12552083	1KDX	HSSP	
P16220	304	12552083	1KDX	HSSP	
Q15788	732	12529333			
Q15788	774	12529333			
P49715	161	12511558	1NWQ	HSSP	
P55854	11	12506199	1WZ0	SMR	
P56817	275	12506199	1FKN	56-446	O
P11387	103	11709553			
P11387	117	11709553<br>12439742			
P11387	153	11709553<br>12439742			
P11387	328	11709553	1A31	175-689	C
P11387	436	11709553	1A31	175-689	C
Q13547	444	11960997<br>12393750	1C3P	HSSP	
Q13547	476	11960997<br>12393750	1C3P	HSSP	
P27540	245	12354770			
O15169	857	12223491			
O15169	860	12223491			
Q9NSC2	1086	12200128	1SP2	HSSP	
P10275	386	12177000			
P10275	520	12177000			
P49716	120	12161447	1H89	HSSP	
Q9UER7	630	12150977			
Q9UER7	631	12150977			

P04150	277	12144530			
P04150	293	12144530			
P04150	703	12144530	1NHZ	500-777	N
P06786	1220	12086615			
P06786	1246	12086615			
P06786	1277	12086615			
P05549	10	12072434			
Q92481	10	12072434			
Q15596	239	12060666			
Q15596	731	12060666			
Q15596	788	12060666			
P56524	559	12032081<br>16166628			
Q13569	330	11889051<br>15823533<br>15959518	1WYW	112-339	C
P04637	386	11867732	1DT7	367-388	H
P05627	229	10788439<br>11867732<br>16055710	1FOS	HSSP	
P05627	257	16055710	1FOS	HSSP	
P46061	526	11853669<br>9442102	1KPS	420-589	O
P23497	297	11792325			
P15330-2	382	11756545	1BVO	HSSP	
P27782	25	11731474			
P27782	267	11731474			
P29590	65	9756909<br>12149243	1BOR	49-104	C
P29590	160	9756909<br>11413191<br>11704854<br>12149243			
P29590	487	9756909<br>11413191<br>11704854<br>12149243			
P29590	490	9756909<br>12149243			
O15350	627	10961991<br>12149243			
P32458	412	12149243			
Q07657	426	12149243			

Q07657	437	12149243			
P19893	175	10684265<br>11264375<br>12149243			
P19893	180	10684265<br>11264375<br>12149243			
P03116	514	11005821<br>12149243			
P13202	450	11602710			
Q9SUI1	258	11581165	1MBK	HSSP	
P03243	104	11553772			
Q64127	724	11313457	1MM2	HSSP	
Q64127	742	11313457	1MM2	HSSP	
O00541	517	11071894			
Q6XA64	802	15105549			
P03206	12	11160742<br>16112644			
P03209	19	15229220			
P03209	213	15229220			
P03209	517	15229220			
P61086	13	15723079	1YLA	1-199	C
P45448	213	15713642			
P45448	289	15713642			
P33244-2	418	15713642	2NLL	HSSP	
P57682	10	15684403	1SP2	HSSP	
P57682	198	15684403	1SP2	HSSP	
Q15744	121	15661739	1H89	HSSP	
Q16514	19	15637059			
Q15542	14	15637059	1ERJ	HSSP	
P04591	474	15613319			
P00445	18	15596868	1B4L	1-153	C
P00445	69	15596868	1B4L	1-153	O
P05750	211	15596868			
P16649	270	15596868			
Q07979	322	15596868			
Q07979	328	15596868			
P04456	60	15542864	1K5Y	59-135	P
P11978	1128	15542864			
Q04322	498	15542864			
P21538	807	15542864	1GVD	HSSP	
Q14814	439	15743823<br>16166628	1EGW	HSSP	

P41970	162	15580297	1HBX	HSSP	
Q9U1H5	438	15788563	1GT0	HSSP	
Q13422	58	15767674	1BBO	HSSP	
Q13422	241	15767674	1BBO	HSSP	
Q9H2X6	32	15766567	1H0V	HSSP	
Q9H2X6	1191	12149243	1H0V	HSSP	
P06400	720	15806172	1GUX	636-787	C
P21063	95	15800065	2AFP	HSSP	
O00180	274	15820677			
P54253	16	15824120			
P54253	194	15824120			
P54253	610	15824120	1OA8	563-694	O
P54253	697	15824120			
P54253	746	15824120			
P54132	317	15829507			
P54132	331	15829507			
P54132	344	15829507			
P54132	347	15829507			
Q8N2W9	35	15831457			
P42575	77	15882978			
P41161	89	15857832	2STT	HSSP	
P41161	263	15857832	2STT	HSSP	
P41161	293	15857832	2STT	HSSP	
P41161	350	15857832	2STT	HSSP	
O60315	391	16061479	1A1G	HSSP	
O60315	866	16061479	1A1G	HSSP	
P01100	265	16055710			
O92597	158	16014952			
P14921	15	16319071			
Q90YL1	61	16256735	1GT0	HSSP	
Q90YL1	365	16256735	1GT0	HSSP	
Q8AXX8	52	16256735	1GT0	HSSP	
Q8AXX8	341	16256735	1GT0	HSSP	
P18412	54	16306045			
Q92793	998	16287980			
Q92793	1033	16287980			
Q92793	1056	16287980			
Q16621	368	16287851			
P40381	103	16168376			
O60016	109	16168376			
O60016	160	16168376			
O42934	198	16168376	1KNA	HSSP	

Q8N4C6	1641	16154161	1DIP	HSSP	
Q8N4C6	1680	16154161	1DIP	HSSP	
Q01543	67	16148010			
Q99683	535	16142216			
Q99683	1083	16142216			
Q99683	1114	16142216			
P37231-2	365	16127449	1FM6	234-505	C
P55265	418	16120648			
O00327	259	16109848	1AM9	HSSP	
Q969V6	499	16098147			
Q969V6	576	16098147			
Q969V6	624	16098147			

		15 proteins within 28 sites [h)]			
Q5U0M2	15	16862185			
Q5U0M2	227	16862185			
Q86YP4	30	16738318			
Q86YP4	487	16738318			
Q5VUR2	33	16738318			
O95600	67	16617055	1SP2	HSSP	
Q07666	96	16568089			
Q13426	210	16478998	1IK9	1-213	C
Q06413	391	16478538	1EGW	HSSP	
P10636-8	339	16464864			
Q9NRA1	314	16443219	1NT0	HSSP	
P49792	2571	16194093			
P49792	2592	16194093			
P49792	2650	16194093	1Z5S	2631-2711	C
P49792	2723	16194093			
P49792	2725	16194093			
P63165	16	16194093	1A5R	1-101	H
P63165	37	16194093	1A5R	1-101	H
P63165	39	16194093	1A5R	1-101	C
P63165	46	16194093	1A5R	1-101	H
P61956	5	16194093	1WZ0	1-93	H
P61956	11	16194093	1A5R	1-93	C
P61956	42	16194093	1A5R	1-93	C
P06876	499	16162816			
P06876	523	16162816			
P12004	164	15931174	1AXC	1-261	C
O15151	254	15907800			

O15151	379	15907800

a) All protein access numbers are based on those in Uniprot KB;
b) SUMO Pos. is the position of sumoylated sites in corresponding sequences.
c) Ref. PMID: Corresponding reference PMID that reported the experimentally identified SUMOylated sites;
d) PDB acc.: protein structure access number in PDB database;
e) Range: Sequence range containing PDB structure;
f) PDB Stru.: PDB structure in corresponding sumoylated sites;
g) The first dataset including 144 substrate sequences (240 sites) reported before Dec. 10 2005;
h) The second dataset including 15 substrate sequences (28 sites) reported between Dec. 10 2005 and Aug. 10 2006;
